# Effect of Rumen-Protected L-Tryptophan or L-Ascorbic Acid on Plasma Metabolites and Milk Production Characteristics of Lactating Holstein Cows during Summer Conditions

**DOI:** 10.3390/ani14121820

**Published:** 2024-06-19

**Authors:** Young-Lae Kim, So-Hee Lee, Gi-Hwal Son, Jong-Suh Shin, Min-Ji Kim, Byung-Ki Park

**Affiliations:** 1 Department of Animal Science, Kangwon National University, Chuncheon 24341, Republic of Korea; dudfo123@naver.com (Y.-L.K.); seesohev@naver.com (S.-H.L.); oscar@naver.com (G.-H.S.); jsshin@kangwon.ac.kr (J.-S.S.); 2 Nonghyup Livestock Research Center, Anseong 17558, Republic of Korea

**Keywords:** Holstein cows, L-tryptophan, L-ascorbic acid, heat stress, milk production characteristics

## Abstract

**Simple Summary:**

Summer heat stress adversely affects the productivity and economic viability of lactating Holstein cows. We explored the effects of dietary supplementation with L-tryptophan or L-ascorbic acid on milk production characteristics and plasma metabolites in lactating Holstein cows during the summer. The results showed that the supplementation with L-tryptophan or L-ascorbic acid reduced heat stress in lactating Holstein cows during the summer. In particular, L-tryptophan supplementation increased the concentration of melatonin in milk. However, there were no significant differences in milk yield or composition between the treatments. Therefore, further research is needed to explore the effects of increasing the dosage of supplements and to investigate the combination strategies of the dietary supplements used in the current study.

**Abstract:**

This study investigated the effects of rumen-protected L-tryptophan or L-ascorbic acid supplementation on the productivity of lactating Holstein cows during a high-temperature period. Thirty cows were assigned to three dietary groups: control (CON), treatment 1 (TRT 1; rumen-protected L-tryptophan, 20 g/cow/d), and treatment 2 (TRT 2; rumen-protected L-ascorbic acid, 20 g/cow/d). As the high-temperature period progressed, the decrease in milk yield and dry matter intake (DMI) in the TRT 1 and TRT 2 groups was lower than that in the CON group. The total protein level in the plasma of the TRT 1 group was higher than that in the CON group (*p* < 0.05). Milk melatonin concentration was higher in the TRT 1 group than in the CON and TRT 2 groups (*p* < 0.05). Thus, the present results indicate that rumen-protected L-tryptophan or L-ascorbic acid has positive effects in preventing declines in DMI and milk yield by reducing heat stress in Holstein cows. In particular, rumen-protected L-tryptophan is considered effective in increasing the melatonin concentration in milk.

## 1. Introduction

Heat stress causes physiological responses, such as increased rectal and body temperatures and heart and respiratory rates, which negatively affect livestock production and economic efficiency [1]. Dairy cows are highly susceptible to heat stress, periods of increased body metabolism, and heat production for milk production [2]. This is because of reduced feed intake, resulting in insufficient nutrients for milk production, which reduces milk yield and quality [2]. Continued efforts are required to reduce heat stress as high-temperature periods and increases in average temperatures are expected owing to global warming.

Neurotransmitters and hormones respond to stress; therefore, dietary supplementation with functional substances capable of controlling stress can be used to reduce stress [3]. Tryptophan is a precursor of serotonin, a monoamine neurotransmitter, and melatonin [4]. Serotonin inhibits substances promoted by stress related to the hypothalamus–pituitary–adrenal cortex axis and alleviates biochemical and physiological occurrences [5]. Melatonin is involved in biorhythm control, immunity, and anti-inflammation, and protects cells from oxidative stress because of its potent antioxidant activity [6]. Regarding this function, tryptophan supplementation has positive effects in reducing increased body and rectal temperatures caused by heat stress [7].

Ascorbic acid reduces stress by promoting the production of gamma-aminobutyric acid and serotonin, which are important neurotransmitters in the central nervous system [8], and by regulating cortisol concentrations [9]. Tryptophan and ascorbic acid play important functional roles in reducing high-temperature stress [10,11]. In particular, tryptophan is designated as an essential amino acid in dairy cows [12] and ascorbic acid requires additional supplementation, as its synthesis may be reduced in high-temperature stress environments [13]. However, rumen microorganisms can destroy these functional substances. Therefore, coatings that bypass the rumen are required for ruminants to effectively absorb and utilize functional substances [12].

Therefore, we hypothesized that supplementation with rumen-protected L-tryptophan or L-ascorbic acid would improve milk yield and quality by reducing heat stress in lactating Holstein cows in high-temperature environments during summer. The present study aimed to investigate the effects of rumen-protected L-tryptophan or L-ascorbic acid supplementation on milk production, milk components, melatonin concentration, plasma metabolites, and stress hormones in lactating Holstein cows during the summer.

## 2. Materials and Methods

The animal experiments were performed at a dairy farm in Pocheon, Gyeonggi. The protocols involving the experimental animals were approved by the ethical and scientific guidelines of the Animal Experiment Ethics Committee of Kangwon National University (approval number: KW-220916-1).

### 2.1. Period

**Figure 1:**
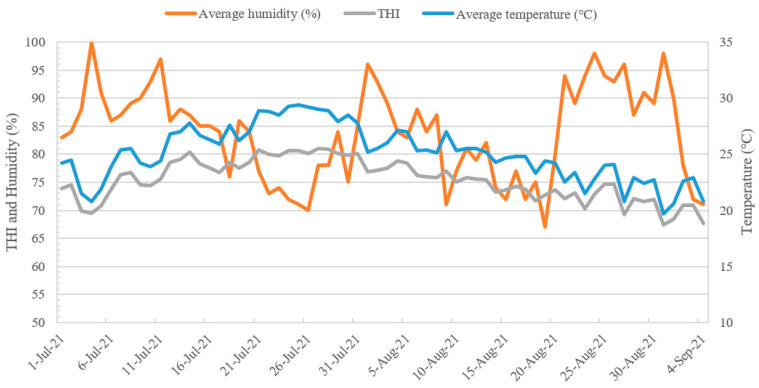
Changes in humidity, temperature–humidity index (THI), and temperature for the entire experimental period.

The present study was conducted from July to September when average temperatures are highest throughout the year (average temperature: 25.1 °C; average maximum temperature: 30.2 °C; average minimum temperature: 21.1 °C; average humidity: 83.8%; average temperature–humidity index [THI]: 75.3). The changes in temperature, humidity, and THI during the investigation period are shown in Figure 1.

### 2.2. Animals, Treatments, and Management

This study included 30 lactating Holstein cows. The thirty cows were assigned to three dietary groups based on milk yield, days in milk, age in months, and parity (Table 1). The control (CON) group was fed a basal diet (total mixed ration [TMR] + concentrate + tall fescue straw), the treatment 1 (TRT 1) group was fed a basal diet + 20 g rumen-protected L-tryptophan (NUVO B&T, Seoul, Republic of Korea), and the treatment 2 (TRT 2) group was fed a basal diet + 20 g rumen-protected L-ascorbic acid (NUVO B&T, Seoul, Republic of Korea). Supplementation levels were based on cow per day. L-tryptophan or L-ascorbic acid was supplemented by top dressing from July to September. The addition of L-tryptophan was set at an intermediate level of 20 g (500 mg/kg of dry matter; considering differences in coating ratios), within the effective range of 15–30 g (including the coating material), as reported by Choi et al. [7]. In previous studies [14,15], L-ascorbic acid was supplemented at 20–30 g (500 mg/kg of dry matter); therefore, a similar level was used in the present study. The rumen-protected L-tryptophan used in the present study comprised 40% L-tryptophan + 60% rumen-protected fat, and rumen-protected L-ascorbic acid comprised 40% L-ascorbic acid + 60% rumen-protected fat. The fat used to coat L-tryptophan or L-ascorbic acid was prepared by mixing palm oil and calcium. The TMR and tall fescue straw were supplied twice daily (07:00 and 18:00) at 15 and 0.5 kg/cow, respectively. The concentrate was supplied using an automatic feeder (automatic feeder, Orion Korea, Pyeongtaek, Republic of Korea) according to the milk yield and nutrient requirements of the NRC [12]. Water and sodium bicarbonate were freely available, and other feeding management practices were based on farm practices. The chemical compositions of the experimental diets are shown in Table 2.

### 2.3. Milk Yield, Feed Intake, and Feed Efficiency

Milk yield was measured thrice (July, August, and September) during the experimental period using a portable milk meter (WB Ezi-Test, TRU-TEST, Auckland, New Zealand) at 6 am and 6 pm, and the average production was calculated by summing the amount of milk produced per day. Feed intake (TMR and tall fescue straw) was calculated by determining the difference between the feed amount before the morning milking and the residual amount before the next morning milking. Concentrate intake was recorded using an automatic feeder (automatic feeder, Orion Korea, Pyeongtaek, Republic of Korea). Feed efficiency was calculated from the milk yield and dry matter intake (DMI).

### 2.4. Milk Components, Fatty Acids, and Melatonin

Milk fat, protein, solid non-fat (SNF), milk urea nitrogen (MUN), and somatic cell count (SCC) were analyzed using an automatic milk component analyzer (Automatic IR 4000/5000 Milk Analyzer, Foss, Hillerod, Denmark), and 4% fat-corrected milk (FCM) was calculated using the following formula:

4% FCM = 0.4 × milk yield (kg/d) + 15 × milk yield (kg/d) × milk fat (%)/100

Fatty acids include saturated fatty acids (SFAs), unsaturated fatty acids (UFAs), monounsaturated fatty acids (MUFAs), and polyunsaturated fatty acids (PUFAs). The fatty acid analysis was performed according to the methods described by Morrison and Smith [16]. Briefly, 20 mL of milk and 200 mL of a 1:2 mixture of methanol and chloroform were mixed, vortexed, and filtered through filter paper. The weight of this filtered solution was equalized with distilled water, and after centrifugation at 1250× *g* for 15 min, the distilled water was removed from the upper layer. The solution with distilled water removed was filtered through a round flask with the addition of 1 g of anhydrous sodium sulfate (Na_2_SO_4_ = 142.04) using filter paper, and the solvent was evaporated at 50 °C using a rotary evaporator (HS-2000NS, Hanshin Scientific CO., Gimpo, Republic of Korea). To analyze fatty acid methyl esters, 1 mL of 0.5 N methanolic sodium hydroxide (NaOH) was added to 1 µL of the sample, heated in boiling water for 15 min, and cooled. Next, 2 mL of Boron Trifluoride (BF_3_, 14%) was added, followed by heating in boiling water for 15 min and cooling. Next, 1 mL of heptane and 2 mL of saturated sodium chloride (NaCl) solution were added to the cooled solution, vortexed, and left undisturbed for 30 min. Subsequently, the supernatant was placed in a tube and evaporated using nitrogen gas, and 1 µL was injected into a gas chromatographer (GC [ACEM 6000 model, Youngin Scientific, Seoul, Republic of Korea]) for fatty acid analysis. The GC conditions were as follows: column, Omegawax 320 capillary column (30 m × 0.32 mm × 0.25 µm, Agilent, Santa Clara, CA, USA); carrier gas, nitrogen (1 mL/min); injection port temperature, 240 °C; detector temperature, 250 °C; oven temperature, 160 °C; and split ratio, 10:1.

Melatonin was analyzed considering the specified experimental procedure using an enzyme-linked immunosorbent assay (ELISA) Kit (OKEH02566, Aviva Systems Biology, San Diego, CA, USA).

### 2.5. Plasma Metabolites and Cortisol

The blood samples were collected at the jugular vein between milking and feeding (7 a.m.) in July, August, and September, and put in a 10 mL vacutainer (Becton Dickinson Co., Franklin Lakes, NJ, USA) with heparin and stabilized at 4 °C for 6 h. After centrifugation at 1250× *g* for 15 min using a centrifuge (UNION 32R, Hanil Science Industrial, Gimpo, Republic of Korea), the supernatant liquid (plasma) was collected, divided into two microtubules, and stored at −80 °C until analysis.

The plasma metabolites were analyzed for glucose (GLU), non-esterified fatty acid (NEFA), blood urea nitrogen (BUN), total protein (TP), albumin (ALB), calcium (Ca), inorganic phosphate (IP), magnesium (Mg), cholesterol (CHOL), gamma-glutamyl-transferase (γ-GTP), and aspartate aminotransferase (AST) using an automatic analyzer (Hitachi 7020, Hitachi Ltd., Tokyo, Japan), and plasma cortisol was analyzed using an ELISA Kit (OKEH02541, Aviva Systems Biology, San Diego, CA, USA).

### 2.6. Statistical Analysis

All the results of the present study are presented as averages and standard errors using IBM SPSS (Statistical Package for the Social Science ver. 29., SPSS Inc., Chicago, IL, USA), and a two-way repeated-measures analysis of variance was performed on the average for each treatment. A post hoc test was performed using Tukey’s method. Differences were considered statistically significant at *p* < 0.05.

## 3. Results

### 3.1. Milk Yield, Feed Intake, and Feed Efficiency

The effects of the rumen-protected L-tryptophan or L-ascorbic acid supplementation on the feed intake and efficiency in the lactating Holstein cows during the high-temperature period are shown in Table 3. The DMI values throughout the experimental period did not differ from those recorded during the same month. However, as the high-temperature period progressed, the decline in feed intake in the TRT 1 and TRT 2 groups was less than that in the CON group. Feed efficiency did not differ throughout the experimental period. Concentrate intake and DMI decreased in all the treatments as the experiment progressed (*p* < 0.05); however, the reduction was lower in the TRT 1 and TRT 2 groups compared to the CON group. Feed efficiency decreased in all the treatments as the experiment continued (*p* < 0.05), but there was no significant difference between the treatments.

### 3.2. Plasma Metabolite and Cortisol

The effects of the rumen-protected L-tryptophan or L-ascorbic acid dietary supplementation on the plasma metabolite and cortisol concentrations in the lactating Holstein cows during the high-temperature period are shown in Table 4. The TP concentration in the TRT 1 group was higher than that in the CON group (*p* < 0.05). There were no differences in the plasma metabolites related to GLU, NEFA, BUN, TP, ALB, Ca, IP, Mg, CHOL, γ-GTP, and AST between the treatment groups throughout the experiment period. The plasma cortisol concentrations tended to increase in the CON group as the high-temperature period progressed, whereas a decreasing trend was observed in September for both the TRT 1 and TRT 2 groups. Furthermore, the plasma cortisol concentrations were affected by the TRT × month interaction (*p* < 0.05).

### 3.3. Milk Yield, Components and Melatonin

**Figure 2:**
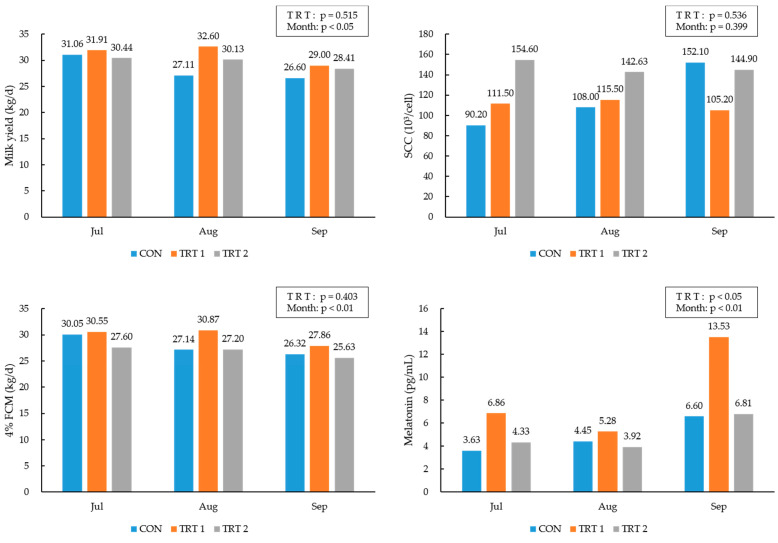
Effects of rumen-protected L-tryptophan or L-ascorbic acid on milk yield, SCC, 4% FCM, and melatonin concentrations of lactating Holstein cows.

The effects of feed supplementation with rumen-protected L-tryptophan or L-ascorbic acid on the milk components of the lactating Holstein cows during the high-temperature period are presented in Figure 2 and Supplementary Table S1. The milk components did not differ among the treatment groups. Although the SCC slightly increased in the CON group, it decreased in the TRT 1 and TRT 2 groups. FCM was decreased in all the treatment groups during the high-temperature period, with a relatively smaller decrease in the TRT 1 group than in the CON and TRT 2 groups. Melatonin concentrations were higher in the TRT 2 group than in the CON and TRT 1 groups (*p* < 0.05) and were higher in September than in July as time progressed (*p* < 0.05).

### 3.4. Fatty Acid

The effects of supplementing with rumen-protected L-tryptophan or L-ascorbic acid on the milk fatty acids of the lactating Holstein cows during the high-temperature period are shown in Supplementary Table S2 and Table 5. Although there were no significant differences, the SFA and UFA ratios (UFA/SFA) tended to increase in all the treatment groups as the temperature increased. In August, when the THI was highest, there was an overall decrease in the PUFA levels. In September, the PUFA levels tended to increase in the CON group but tended to decrease in the TRT 1 and TRT 2 groups.

## 4. Discussion

Tryptophan is a precursor for the synthesis of serotonin and melatonin [4] and improves heat dissipation by increasing skin temperature and reducing heat stress by regulating body temperature [17]. Tryptophan in the blood penetrates the brain through the blood vessel–brain barrier and is converted to serotonin through 5-hydroxytryptophan in the pinealocytes, and serotonin is converted to melatonin by N-acetyltransferase [18]. Kollmann et al. [19] reported that in ruminants, tryptophan and melatonin concentrations in the blood were increased by the dietary supplementation with rumen-protected L-tryptophan, and milk melatonin concentration was affected by blood melatonin concentration. In the present study, we observed an increase in the melatonin concentration in milk following the dietary supplementation with rumen-protected L-tryptophan, which reduced stress by increasing the serotonin and melatonin synthesis. In addition, the special coatings suggest that ruminants can effectively use L-tryptophan without breakdown by the rumen microbes.

BUN, the final product of protein metabolism, increases liver and kidney dysfunction [20] and it is positively correlated with high temperature and humidity [21,22]. The results of the present study are consistent with those of Farombi and Onyema [23], who revealed that vitamin C supplementation reduced BUN concentration by improving oxidative stress. Furthermore, it is thought to be the cause of decreased AST and γ-GTP concentrations and evaluation indices for liver function in the L-ascorbic acid treatment group. In the present study, plasma BUN concentrations in the TRT 1 and TRT 2 groups decreased more than those in the CON group in September compared to those in August due to the antioxidant effects of the L-tryptophan or L-ascorbic acid supplementation. TP is another blood metabolite that represents protein metabolism. TP concentration in the plasma increased as protein intake increased [20]. In the present study, the concentrate intake in the TRT 1 group tended to be higher than that in the CON group during August and September. Accordingly, plasma TP concentration in the TRT 1 group was higher than that in the CON group (*p* < 0.05).

In the present study, cortisol levels were used as an indicator of stress. The cortisol and epinephrine concentrations increased in the plasma of stressed animals [5]. Minton [5] reported that cortisol inhibits reproductive, immune, and digestive functions. Dietary supplementation with ascorbic acid reduces cortisol release and controls the body temperature by acting as an inhibitory neurotransmitter in the hypothalamus [24]. In the present study, the plasma cortisol concentrations were lower in the TRT 1 and TRT 2 groups than in the CON group, which may be due to the stress-reducing effects of the rumen-protected L-tryptophan or L-ascorbic acid.

Oxidative stress in the mammary glands in a high-temperature environment increases the incidence of mastitis and SCC, with higher SCC in milk produced during the summer season than in milk produced during the spring season [25,26]. However, in the present study, the SCC of the TRT 1 and TRT 2 groups did not increase during the high-temperature period, which was attributed to the antioxidant effects of L-tryptophan or L-ascorbic acid.

The MUN concentration is related to the energy to crude protein (CP) ratio, rumen CP decomposition rate, and CP intake [26]. In the present study, the MUN concentration increased in all the treatment groups as the high-temperature period progressed. However, in September, it decreased only in the L-ascorbic acid treatment group compared to August. The liver utilizes ammonia and urea to metabolize protein, and the kidneys remove toxins such as ammonia and urea from the blood. Therefore, a diet that overfeeds the protein sources needed for growth and milk production puts a strain on the liver and kidneys, especially during summer heat stress [27,28]. L-ascorbic acid is depleted when exposed to heat stress and is known to influence damage suppression, functional protection, and recovery under oxidative stress, and it is also known to deplete rapidly when exposed to heat stress [27]. The author mentions that L-ascorbic acid antioxidants improve liver and kidney function. In the present study, the MUN levels did not increase in the TRT2 group during September, which was the period of the highest heat stress, and it was thought that L-ascorbic acid antioxidants improved liver and kidney function.

Heat stress is a major factor affecting milk yield due to the decrease in DMI [2]. Baumgard et al. [29] reported that the DMI and milk yield of lactating Holstein cows under heat stress decreased by 5 and 6 kg/day, respectively. In the present study, milk yield decreased in all the groups as the study period progressed because of the decrease in concentrate intake caused by heat stress. However, considering that the TRT 1 and TRT 2 groups showed a relatively smaller decrease in milk yield than the CON group, it was considered that the rumen-protected L-tryptophan or L-ascorbic acid had a positive effect on stress reduction. According to Choi et al. [7], the supplementation with rumen-protected L-tryptophan in lactating Holstein cows increased the DMI and milk yield when the THI exceeded 80. Tanaka et al. [30] reported that milk yield was higher in a high ascorbic acid concentration group than in a low ascorbic acid concentration group. Kumar et al. [31] reported that DMI increased with melatonin supplementation.

In the present study, blood glucose concentration was higher in the treatment groups than in the CON group. The dietary supplementation or injection of tryptophan into ruminants increased blood melatonin and cholecystokinin (CCK) concentrations, and plasma glucose (GLU) levels, likely due to the activity of CCK on α-amylase [6,7]. Initially, we hypothesized that the plasma GLU concentration in the TRT 1 group would increase due to the rise in DMI, CCK, and α-amylase. However, milk yield, DMI, and GLU did not increase with the supplementation of rumen-protected L-tryptophan or L-ascorbic acid in this study. The observed difference may be related to the supplementation level of L-tryptophan or L-ascorbic acid. The energy balance of dairy cows affects the fatty acids in milk [32]. The increase in body temperature and decrease in feed intake due to heat stress result in a negative energy balance [2]. Furthermore, body fat is mobilized to synthesize milk fat, which increases the UFA ratio of milk but decreases the SFA ratio [33]. Bohlouli et al. [34] reported that dairy cows mobilize body fat through a negative energy balance at a high THI. In the present study, UFA and PUFA tended to increase as high-temperature stress persisted in all the treatments; however, the increase was not statistically significant.

Our results indicated that supplementation with L-tryptophan and L-ascorbic acid mitigated heat stress-induced reductions in dairy cattle productivity, such as milk yield, milk composition, and somatic cell count, and also reduced cortisol levels, an indicator of stress. However, the present study did not measure rectal temperature and respiratory rate, which is a limitation in evaluating whether rumen-protected L-tryptophan or L-ascorbic acid could alleviate the physiological responses to heat stress. Future studies are needed to evaluate the direct effects of these supplements on heat stress by including measurements of rectal temperature and respiratory rate.

## 5. Conclusions

Our findings of this study indicate that rumen-protected L-tryptophan or L-ascorbic acid has positive effects on preventing declines in feed intake and milk yield by reducing heat stress (decrease in plasma cortisol concentration) in lactating Holstein cows during summer. In particular, rumen-protected L-tryptophan is considered effective in increasing the melatonin concentration in milk. However, further studies are required to investigate the efficacy of various supplementation levels of these additives, their simultaneous supply, or supplementation prior to the onset of heat stress.

## Figures and Tables

**Table 1:** Milk yield, days in milk, months old, and parity of Holstein cows.

Items	CON	TRT 1 ^1^	TRT 2 ^2^
Yield	31.06 ± 5.70	31.91 ± 8.08	30.44 ± 6.84
Days in milk	226.40 ± 96.83	217.78 ± 83.56	212.80 ± 84.98
Months old	60.50 ± 34.26	54.30 ± 27.48	56.11 ± 23.54
Parity	2.60 ± 2.46	2.40 ± 1.35	2.90 ± 2.23

^1^ T1: rumen-protected L-tryptophan treatment; ^2^ T2: rumen-protected L-ascorbic acid treatment.

**Table 2:** Chemical composition of experimental diets (dry matter basis).

Items	Concentrate	TMR ^1^	Tall Fescue Straw
Dry matter (%)	93.69 ± 0.26	65.74 ± 0.34	84.60 ± 0.24
Crude protein (%)	21.01 ± 0.22	24.28 ± 0.10	4.87 ± 0.04
Ether extract (%)	6.80 ± 0.31	3.21 ± 0.54	0.99 ± 0.10
Crude ash (%)	7.57 ± 0.30	11.50 ± 0.03	10.44 ± 0.78
Crude fiber (%)	12.78 ± 0.39	28.81 ± 0.75	50.01 ± 0.54
Neutral detergent fiber (%)	48.61 ± 0.45	65.56 ± 0.56	93.64 ± 0.42
Acid detergent fiber (%)	25.39 ± 0.29	41.65 ± 0.86	65.35 ± 0.08

^1^ TMR: total mixed ration.

**Table 3:** Effects of rumen-protected L-tryptophan or L-ascorbic acid on feed intake and feed efficiency of lactating Holstein cows.

Items	July			August			September			*p*-Value		
	CON	TRT 1	TRT 2	CON	TRT 1	TRT 2	CON	TRT 1	TRT 2	Month	TRT	TRT × Month
Feed intake (DM kg/d)												
Concentrate	3.85 ± 1.09	4.08 ± 1.41	3.77 ± 1.32	3.21 ± 1.23	4.06 ± 1.44	3.63 ± 1.16	2.98 ± 1.00	3.68 ± 0.92	3.44 ± 0.97	0.030	0.408	0.596
Total mixed ration	19.72	19.72	19.72	19.72	19.72	19.72	19.72	19.72	19.72	-	-	-
Tall fescue straw	0.85	0.85	0.85	0.85	0.85	0.85	0.85	0.85	0.85	-	-	-
DMI ^1^	24.42 ± 1.09	24.57 ± 1.42	24.34 ± 1.32	23.69 ± 1.31	24.55 ± 1.48	24.11 ± 1.25	23.46 ± 1.13	24.16 ± 0.92	23.92 ± 1.13	0.024	0.475	0.587
Feed efficiency (milk yield/DMI, kg/kg)	1.27 ± 0.18	1.29 ± 0.27	1.24 ± 0.21	1.13 ± 0.21	1.31 ± 0.29	1.24 ± 0.22	1.12 ± 0.22	1.20 ± 0.18	1.18 ± 0.17	0.018	0.553	0.693

^1^ DMI: dry matter intake.

**Table 4:** Effects of rumen-protected L-tryptophan or L-ascorbic acid on plasma metabolites and cortisol concentrations of lactating Holstein cows.

Items	July			August			September			*p*-Value		
	CON	TRT 1	TRT 2	CON	TRT 1	TRT 2	CON	TRT 1	TRT 2	Month	TRT	TRT × Month
GLU ^1^ (mg/dL)	63.50 ± 2.95	62.60 ± 3.17	59.20 ± 12.93	60.40 ± 7.38	62.00 ± 4.24	59.44 ± 6.19	58.30 ± 3.20	61.20 ± 5.35	59.56 ± 3.81	0.241	0.325	0.329
NEFA ^2^ (uEq/L)	112.63 ± 11.34	126.67 ± 25.75	128.13 ± 22.43	54.00 ± 28.46	66.56 ± 27.67	74.63 ± 24.14	39.25 ± 15.27	47.00 ± 21.70	46.25 ± 11.46	<0.001	0.232	0.632
BUN ^3^ (mg/dL)	19.74 ± 2.34	18.80 ± 2.92	19.15 ± 3.47	22.85 ± 2.73	24.81 ± 2.86	24.89 ± 2.60	21.50 ± 1.63	22.46 ± 3.26	21.26 ± 1.79	<0.001	0.735	0.471
TP ^4^ (g/dL)	8.43 ± 0.36	8.57 ± 0.48	8.35 ± 0.25	7.34 ± 0.55	8.02 ± 0.58	7.93 ± 1.06	6.46 ± 0.37	7.03 ± 0.58	6.84 ± 0.50	<0.001	0.033	0.191
ALB ^5^ (g/dL)	4.35 ± 0.18	4.29 ± 0.12	4.30 ± 0.26	3.74 ± 0.30	4.00 ± 0.24	3.97 ± 0.35	3.34 ± 0.17	3.57 ± 0.35	3.46 ± 0.21	<0.001	0.097	0.144
Ca ^6^ (mg/dL)	9.65 ± 0.41	9.51 ± 0.34	9.45 ± 0.47	8.51 ± 0.59	8.79 ± 0.46	8.71 ± 1.11	7.56 ± 0.37	7.79 ± 0.67	7.58 ± 0.48	<0.001	0.736	0.267
IP ^7^ (mg/dL)	6.74 ± 0.81	6.64 ± 1.19	6.93 ± 1.25	5.23 ± 1.00	6.17 ± 0.99	6.13 ± 1.05	4.54 ± 0.51	5.22 ± 0.89	4.83 ± 0.88	<0.001	0.280	0.313
Mg ^8^ (mg/dL)	2.44 ± 0.16	2.43 ± 0.19	2.44 ± 0.21	2.49 ± 0.28	2.67 ± 0.29	2.73 ± 0.35	2.16 ± 0.18	2.30 ± 0.22	2.22 ± 0.16	<0.001	0.295	0.348
CHOL ^9^ (mg/dL)	246.40 ± 56.56	258.40 ± 61.91	265.20 ± 64.71	241.60 ± 47.57	272.10 ± 55.56	267.00 ± 44.29	216.70 ± 35.93	254.00 ± 74.15	235.89 ± 47.90	0.066	0.465	0.564
γ-GTP ^10^ (U/L)	20.40 ± 11.51	18.20 ± 4.66	24.00 ± 11.76	37.00 ± 13.98	33.60 ± 7.21	45.67 ± 27.60	30.70 ± 6.80	32.00 ± 7.92	33.11 ± 13.12	<0.001	0.484	0.292
AST ^11^ (U/L)	79.70 ± 21.44	76.00 ± 8.23	81.90 ± 21.87	87.10 ± 27.80	95.60 ± 21.91	101.33 ± 22.98	74.60 ± 15.26	79.80 ± 15.39	76.00 ± 13.11	0.287	0.699	0.227
Cortisol (ng/mL)	35.73 ± 0.59	35.06 ± 1.62	34.81 ± 2.57	36.33 ± 0.74	35.22 ± 1.61	34.80 ± 2.54	36.17 ± 0.52	32.82 ± 3.91	33.28 ± 3.99	0.012	0.192	0.043

^1^ GLU: glucose; ^2^ NEFA: non-esterified fatty acid; ^3^ BUN: blood urea nitrogen; ^4^ TP: total protein; ^5^ ALB: albumin; ^6^ Ca: calcium; ^7^ IP: inorganic phosphate; ^8^ Mg: magnesium; ^9^ CHOL: cholesterol; ^10^ γ-GTP: gamma-glutamyl-transferase; ^11^ AST: aspartate-amino-transferase.

**Table 5:** Effects of rumen-protected L-tryptophan or L-ascorbic acid on fatty acid composition in milk of lactating Holstein cows.

Items	July			August			September			*p*-Value		
	CON	TRT 1	TRT 2	CON	TRT 1	TRT 2	CON	TRT 1	TRT 2	Month	TRT	TRT × Month
SFA ^1^	70.38 ± 3.54	67.99 ± 3.89	66.46 ± 5.06	67.99 ± 2.87	67.38 ± 3.38	67.74 ± 2.84	68.08 ± 2.24	66.70 ± 1.40	65.96 ± 1.55	0.595	0.398	0.248
MUFA ^2^	27.30 ± 2.95	29.44 ± 3.84	30.99 ± 4.72	29.10 ± 2.65	30.01 ± 3.19	29.40 ± 2.67	28.94 ± 1.45	30.74 ± 1.54	31.34 ± 1.34	0.632	0.408	0.267
PUFA ^3^	2.32 ± 0.86	2.56 ± 0.32	2.55 ± 0.56	2.91 ± 0.29	2.61 ± 0.26	2.86 ± 0.51	2.98 ± 1.19	2.57 ± 0.17	2.69 ± 0.28	0.445	0.628	0.088
UFA ^4^	29.62 ± 3.54	32.01 ± 3.89	33.54 ± 5.06	32.01 ± 2.87	32.62 ± 3.38	32.26 ± 2.84	31.92 ± 2.24	33.30 ± 1.40	34.04 ± 1.55	0.595	0.398	0.248
n-6/n-3	7.84 ± 1.06	7.03 ± 1.77	7.77 ± 2.57	7.39 ± 1.22	7.35 ± 0.99	7.50 ± 3.04	8.04 ± 0.92	7.91 ± 1.96	6.88 ± 1.63	0.895	0.985	0.196
UFA/SFA	0.42 ± 0.07	0.48 ± 0.09	0.51 ± 0.13	0.47 ± 0.06	0.49 ± 0.07	0.48 ± 0.06	0.47 ± 0.05	0.50 ± 0.03	0.52 ± 0.04	0.825	0.371	0.241

^1^ SFA: saturated fatty acid; ^2^ MUFA: monounsaturated fatty acid; ^3^ PUFA: polyunsaturated fatty acid; ^4^ UFA: unsaturated fatty acid.

## Data Availability

The original contributions presented in the study are included in the article and supplementary material.

## Supplementary Materials

The following supporting information can be downloaded at: https://www.mdpi.com/article/10.3390/ani14121820/s1, Table S1: Effects of rumen-protected L-tryptophan or L-ascorbic acid on milk composition and melatonin concentrations of lactating Holstein cows.; Table S2: Effects of rumen-protected L-tryptophan or L-ascorbic acid on fatty acid composition in milk of lactating Holstein cows.
