# Supplementary material for: Effect of Rumen-Protected L-Tryptophan or L-Ascorbic Acid on Plasma Metabolites and Milk Production Characteristics of Lactating Holstein Cows during Summer Conditions
Source: Animals (Basel). 2024 Jun 19;14(12):1820. doi: 10.3390/ani14121820 (PMC11200681; doi:10.3390/ani14121820)
Supplement: Supplementary file 1 [file animals-14-01820-s001.zip › animals-3014570-supplementary.pdf]

Table S1. Effects of rumen protected L-tryptophan or L-ascorbic acid on milk composition and melatonin concentrations of lactating Holstein cows.

Items	Jul			Aug			Sep			P-value		
	CON	TRT 1	TRT 2	CON	TRT 1	TRT 2	CON	TRT 1	TRT 2	Month	TRT	TRT × month
Milk yield (kg/d)	31.06 ± 5.70	31.91 ± 8.08	30.44 ± 6.84	27.11 ± 6.55	32.60 ± 9.06	30.13 ± 6.95	26.60 ± 6.41	29.00 ± 5.29	28.41 ± 5.37	0.016	0.515	0.713
Protein (%)	3.25 ± 0.30	3.25 ± 0.22	3.17 ± 0.16	3.39 ± 0.35	3.26 ± 0.30	3.24 ± 0.29	3.46 ± 0.38	3.35 ± 0.28	3.25 ± 0.28	0.675	0.490	0.296
Protein (kg/d)	1.01 ± 0.18	1.03 ± 0.24	0.96 ± 0.19	0.92 ± 0.23	1.05 ± 0.28	0.96 ± 0.17	0.91 ± 0.18	0.97 ± 0.17	0.92 ± 0.16	0.056	0.566	0.810
Fat (%)	3.81 ± 0.57	3.68 ± 0.67	3.49 ± 0.68	4.00 ± 0.66	3.70 ± 0.66	3.43 ± 0.54	4.03 ± 0.88	3.78 ± 0.71	3.43 ± 0.59	0.400	0.214	0.525
Fat (kg/d)	1.18 ± 0.24	1.19 ± 0.41	1.03 ± 0.15	1.09 ± 0.32	1.19 ± 0.34	1.01 ± 0.16	1.05 ± 0.24	1.08 ± 0.23	0.95 ± 0.12	0.016	0.267	0.868
SCC[1] (10^3/Cell)	90.20 ± 70.53	111.50 ± 130.30	154.60 ± 82.38	108.00 ± 90.85	115.50 ± 130.34	142.63 ± 56.94	152.10 ± 137.60	105.20 ± 115.08	144.90 ± 73.33	0.399	0.536	0.201
SNF[2] (%)	9.54 ± 0.32	9.52 ± 0.31	9.40 ± 0.37	9.79 ± 0.48	9.62 ± 0.39	9.59 ± 0.44	9.10 ± 0.41	9.08 ± 0.30	8.96 ± 0.39	< 0.001	0.623	0.989
MUN[3] (mg/dL)	14.59 ± 1.46	14.32 ± 1.11	13.73 ± 1.55	15.75 ± 1.22	15.39 ± 1.15	15.05 ± 1.01	16.73 ± 2.24	16.46 ± 1.52	14.84 ± 1.01	< 0.001	0.101	0.137
4% FCM[4] (kg/d)	30.05 ± 5.41	30.55 ± 9.05	27.60 ± 3.96	27.14 ± 7.05	30.87 ± 8.46	27.20 ± 4.80	26.32 ± 5.`03	27.86 ± 4.99	25.63 ± 3.27	0.008	0.403	0.765
Melatonin (pg/mL)	3.63 ± 3.33	4.33 ± 1.41	6.86 ± 3.53	4.45 ± 1.92	3.92 ± 1.75	5.28 ± 2.23	6.60 ± 1.08	6.81 ± 5.61	13.53 ± 4.01	0.004	0.022	0.240

[1]SCC: somatic cell count; [2]SNF: solid not fat; [3]MUN: milk urea nitrogen; [4]FCM: fat corrected milk

Table S2. Effects of rumen protected L-tryptophan or L-ascorbic acid on fatty acid composition in milk of lactating Holstein cows.

Items	Jul			Aug			Sep			P-value		
	CON	TRT 1	TRT 2	CON	TRT 1	TRT 2	CON	TRT 1	TRT 2	Month	TRT	TRT × month
Octanoic (C8:0, %)	4.87 ±4.41	2.60 ±1.15	2.63 ±2.31	2.11 ±0.79	2.85 ±1.14	2.53 ±1.12	2.06 ±0.41	2.11 ±0.80	1.74 ±0.33	0.110	0.850	0.760
Decanoic (C10:0. %)	3.13 ±0.79	2.58 ±0.57	2.50 ±0.78	2.72 ±0.30	2.48 ±0.31	2.39 ±0.30	3.01 ±0.23	2.73 ±0.36	2.53 ±0.24	0.370	0.262	0.809
Lauric (C12:0, %)	4.61 ±0.58	4.17 ±0.82	4.28 ±1.28	4.48 ±0.42	4.23 ±0.38	4.04 ±0.43	4.85 ±0.29	4.67 ±0.35	4.38 ±0.24	0.125	0.624	0.965
Myristic (C14:0, %)	12.07 ±0.84	11.82 ±1.96	11.72 ±2.60	12.36 ±0.77	11.98 ±0.85	11.85 ±0.92	12.68 ±0.83	12.45 ±0.66	12.11 ±0.32	0.156	0.655	0.925
Palmitic (C16:0, %)	29.68 ±3.38	29.85 ±3.09	30.21 ±2.64	31.63 ±3.40	31.39 ±1.82	32.52 ±3.19	31.23 ±3.97	31.07 ±1.43	31.55 ±1.89	0.126	0.794	0.388
Palmitoleic (C16:1n-9, %)	1.91 ±0.20	2.07 ±0.58	2.20 ±0.44	2.01 ±0.26	1.98 ±0.24	2.02 ±0.18	2.12 ±0.16	2.23 ±0.21	2.19 ±0.17	0.245	0.418	0.151
Stearic (C18:0, %)	13.36 ±2.09	14.24 ±2.01	13.71 ±1.69	13.36 ±1.95	12.87 ±0.69	12.78 ±1.45	12.49 ±1.24	12.59 ±0.88	12.58 ±1.06	0.003	0.335	0.257
Oleic (C18:1n-9, %)	24.61 ±2.77	26.79 ±3.60	28.37 ±4.36	26.86 ±2.62	27.67 ±2.82	27.02 ±2.63	26.65 ±1.59	28.27 ±1.41	28.98 ±1.16	0.510	0.287	0.279
Linoleic (C18:2n-6, %)	2.05 ±0.73	2.22 ±0.26	2.26 ±0.56	2.55 ±0.24	2.30 ±0.26	2.45 ±0.35	2.65 ±1.08	2.28 ±0.14	2.32 ±0.32	0.499	0.727	0.067
Linolenic (C18:3n-3, %)	0.27 ±0.13	0.34 ±0.11	0.30 ±0.04	0.36 ±0.08	0.31 ±0.02	0.41 ±0.29	0.33 ±0.12	0.30 ±0.07	0.35 ±0.05	0.753	0.955	0.591
Arachdic (C20:0, %)	0.81 ±0.56	1.01 ±1.49	0.42 ±0.09	0.53 ±0.14	0.66 ±0.26	0.52 ±0.15	0.54 ±0.20	0.43 ±0.03	0.45 ±0.02	0.178	0.540	0.457
Behenic (C22:0, %)	1.02 ±0.53	0.72 ±0.70	0.44 ±0.26	0.43 ±0.05	0.47 ±0.25	0.68 ±0.54	0.47 ±0.28	0.42 ±0.08	0.40 ±0.04	0.025	0.425	0.203
Erucic (C22:1, %)	0.78 ±0.48	0.58 ±0.64	0.42 ±0.50	0.23 ±0.06	0.36 ±0.31	0.36 ±0.25	0.17 ±0.02	0.21 ±0.08	0.19 ±0.01	0.055	0.583	0.090
Lignoceric (C24:0, %)	0.84 ±0.50	0.99 ±1.23	0.56 ±0.25	0.37 ±0.33	0.45 ±0.13	0.42 ±0.14	0.76 ±0.99	0.24 ±0.11	0.23 ±0.06	0.252	0.469	0.367
